# Receiving the Charge of Governor and President

**Published:** 1920-02-28

**Authors:** 


### THE PRINCE OF WALES at ST. BARTHOLOMEW'S.

### Receiving the Charge of Governor and President.

The Prince of Wales holds the "green staff," which is the symbol of office of the President of the Royal Hospital of St. Bartholomew, resuming the long association that the Royal House has maintained with the oldest of the medical charities of London. His father and grandfather, when themselves Prince, accepted election to the office, which they relinquished only on succeeding to the throne, since which time King George has been Patron. In an earlier age the President took a most active part in affairs, but with the passage of time the post has become more and more one of dignity, the Treasurer—to-day Viscount Sandhurst—being the responsible head of the administration.

It was a hearty gathering that assembled in the Grand Hall at St. Bartholomew's on Thursday of last week for the Prince's installation. A special Court of Governors had been summoned, and on chairs set in a square about the head table the Governors sat. The larger space in the long apartment was filled by a distinguished company of medical men and civic and other supporters of the hospital, the whole of one side being kept for the sisters and nurses. Their garb of mercy, seen thus massed together, gave the required picturesque touch to the scene, with its background of paintings of eminent physicians and surgeons who have served the hospital well and the portrait of King Henry VIII. above the mantelpiece. Here the Tudor monarch who took to himself credit for founding (or refounding) the hospital, because, having seized all its revenues, he returned some of them, looks across the centuries to the canvas of King Edward VII. confronting him. Rahere's memory is kept in more reverence at St. Bartholomew's than that of Henry VIII.

The quadrangle was alive with animation. Many patients in beds and chairs were wheeled out to enjoy the sunshine of a June day cast into February. One missed the hospital blue familar for so many years, which has not been seen since the military activities of Bart's ceased, but many ex-soldiers are undergoing treatment, and a group of them paraded, with whom the Prince of Wales chatted as soon as he had alighted from his car. The Hon. Sir Sidney Greville was in attendance. Nurses cheered, and servants of the staff, hastening up from unknown depths, cheered, and the medical students made up for the paucity of their numbers by the loudness of their welcome—for a coincidence befell that an important football tie was fixed for the same afternoon that the Prince came, and had attracted away not a few devotees.

The cheers came up to the Grand Hall. There, a moment later, the Prince of Wales appeared, escorted by Lord Sandhurst and Sir Ernest Cooper, Lord Mayor. After the first cordial greeting, all was hushed for the business of the Court. Mr. Thomas Hayes, clerk to the Governors, read first to the Prince the Charge of a Governor, that he should acquit himself in that office with all faithfulness and sincerity, endeavouring that the affairs and business of the hospital may be well ordered and managed, "and promoting the weal and advantage of the poor, wounded, sick, maimed, and diseased persons harboured in the same hospital." That was the quaint phraseology used, and it brought back the pleasant flavour of past ages. To this and to the Charge of President, a more formal document, the Prince subscribed. Lord Sandhurst thereupon surrendered to him the chair and the conduct of the Court, and His Royal Highness was invested with the "green staff"—a gold-headed dark cane, such as every doctor of the seventeenth and eighteenth centuries affected when making his rounds.

There were minutes to be signed, and a few new governors, two ladies being among them, to receive their printed Charges. With each of these the Prince shook hands, then rising said:

"My lords, ladies and gentlemen,—There being no other business we will adjourn the Court; and I should like at the same time to say what a privilege it is to take up the office of President, and I am quite looking forward to seeing something of the hospital for myself. Thank you for your very kind reception."

Five minutes sufficed for the little ceremony. The Prince thereafter spent an hour in the hospital, making a tour of all the wards. With the beadle bearing his silver-topped staff going ahead, a small procession was formed, Lord Sandhurst conducting the Prince, and with him were Colonel Howard Tooth, the senior physician; Colonel Sir D'Arcy Power, the senior surgeon; Miss McIntosh, the matron; Mr. Hayes, and Captain C. M. Power, steward of the hospital. Crossing the quadrangle a visit was first paid to the eastern wing. This building was set aside for military use during the war, and to-day it bears a tablet commemorating the reception and treatment there of 5,400 soldiers from the end of 1914 till January 1919. The Lucas ward (female surgical), and operating-theatre, and the Kenton and Harley (surgical), and Rahere and Colston (medical) wards were passed through in turn, the sister and physician and surgeon in each case being presented, and accompanying the Prince round. His Royal Highness exchanged a few words with practically every patient, and asked especially for the ex-soldiers. With a member of the W.A.A.C.s, who had seen service in France and is now recovering health, the Prince talked for some moments.

At the dispensary His Royal Highness was much interested in a tabloid-making machine, which he watched in operation, asking many questions, and he acknowledged surprise at the size of the surgery, in which often eight hundred out-patients are assembled in the morning. A few cordial words before leaving expressed thanks and satisfaction at all he had seen.

